# Safety and efficacy of umbilical cord tissue-derived mesenchymal stem cells in the treatment of patients with aging frailty: a phase I/II randomized, double-blind, placebo-controlled study

**DOI:** 10.1186/s13287-024-03707-2

**Published:** 2024-04-29

**Authors:** Yingqian Zhu, Ce Huang, Liang Zheng, Qingqing Li, Jianli Ge, ShaSha Geng, Miaomiao Zhai, Xin Chen, Huixiao Yuan, Yang Li, Wenwen Jia, Keping Sun, Yan Li, Tong Ye, Zhengmei Zhao, Hailiang Liu, Zhongmin Liu, Hua Jiang

**Affiliations:** 1 Department of Geriatrics, Shanghai East Hospital, Tongji University School of Medicine, Shanghai, 200120 China; 2 Department of General Medicine, Shanghai East Hospital, Tongji University School of Medicine, Shanghai, 200120 China; 3 Institute for Regenerative Medicine, Shanghai East Hospital, Tongji University School of Medicine, Shanghai, 200120 China; 4 Institutes of Biomedical Sciences, Fudan University, Shanghai, 200032 China; 5 Shanghai Institute of Infectious Disease and Biosecurity, Fudan University, Shanghai Municipality, 200032 China; 6 Research Center for Translational Medicine, Shanghai East Hospital, Tongji University School of Medicine, Shanghai, 200120 China; 7 Shanghai Institute of Stem Cell Research and Clinical Translation, Shanghai, 200120 China; 8 Department of Cardiovascular Surgery, Shanghai East Hospital, Tongji University School of Medicine, Shanghai, 200120 China; 9 Translational Medical Center for Stem Cell Therapy, Shanghai East Hospital, Tongji University, Shanghai, 200120 China

**Keywords:** Aging frailty, Human umbilical cord derived mesenchymal stem cells, Safety and efficacy, Randomized controlled trial, Quality of life, physical performance, Chronic inflammation

## Abstract

**Background:**

Mesenchymal stem cells (MSCs) hold a great promise for cell-based therapy in the field of regenerative medicine. In this study, we aimed to evaluate the safety and efficacy of intravenous infusion of human umbilical cord-derived MSCs (HUC-MSCs) in patients with aging frailty.

**Methods:**

In this randomized, double-blind, placebo-controlled trial, participants diagnosed with aging frailty were randomly assigned to receive intravenous administrations of HUC-MSCs or placebo. All of serious adverse events and AEs were monitored to evaluate the safety of treatment during the 6-month follow-up. The primary efficacy endpoint was alteration of physical component scores (PCS) of SF-36 qualities of life at 6 months. The secondary outcomes including physical performance tests and pro-inflammatory cytokines, were also observed and compared at each follow-up visits. All evaluations were performed at 1 week, 1, 2, 3 and 6 months following the first intravenous infusion of HUC-MSCs.

**Results:**

In the MSCs group, significant improvements in PCS of SF-36 were observed from first post-treatment visit and sustained throughout the follow-up period, with greater changes compared to the placebo group (*p* = 0.042). EQ-VAS scores of MSCs group improved significantly at 2 month (*p* = 0.023) and continued until the end of the 6-month visit (*p* = 0.002) in comparison to the placebo group. The timed up and go (TUG) physical performance test revealed significant group difference and showed continual enhancements over 6 months (*p* < 0.05). MSC transplantation improved the function of 4-m walking test (4MWT) compared with the placebo group with a decrease of 2.05 s at 6 months of follow-up (*p* = 0.21). The measurement of grip strength revealed group difference with MSCs group demonstrating better performance, particularly at 6 months (*p* = 0.002). Inflammatory cytokines (TNF-α, IL-17) exhibited declines in MSCs group at 6 months compared to the placebo group (*p* = 0.034 and 0.033, respectively). There was no difference of incidence of AEs between the two groups.

**Conclusion:**

Intravenous transplantation of HUC-MSCs is a safe and effective therapeutic approach on aging frailty. The positive outcomes observed in improving quality of life, physical performance, and reducing chronic inflammation, suggest that HUC-MSC therapy may be a promising potential treatment option for aging frailty.

*Trial Registration*: Clinicaltrial.gov; NCT04314011; https://clinicaltrials.gov/ct2/show/NCT04314011.

**Supplementary Information:**

The online version contains supplementary material available at 10.1186/s13287-024-03707-2.

## Background

With the progressive increase in the elderly population, aging frailty is becoming a major public health problem worldwide [1]. Frailty is a common geriatric syndrome in the aging process and represents as a state of heightened vulnerability to potential stressors as a consequence of reduction in physiological reserves across multiple systems [2]. Frailty has various causes and contributors, culminating in the diminished strength, endurance and cumulative deterioration of physical functions in aging individuals, which amplifies the risk of hospitalization, disability, and mortality [3, 4]. Furthermore, older patients with comorbidities, particularly those have cardiovascular and neuropsychiatric disease are more susceptible to developing frailty [5]. The overall prevalence of aging frailty in community-dwelling population was 6.9%, which is notably higher in older females with lower levels of education and income [3]. In the context of aging population, frailty becomes more prominent among older adults. It was estimated that frailty prevalence increased substantially from 3.9 to 25% in individuals aged 65 and over, with a more pronounced impact on adults aged 85 or older [6]. The escalating prevalence in the aging population, coupled with the associated adverse events, has transformed frailty into a global public health challenge, imposing significant burdens on human health, as well as the economic and social fields [7]. Consequently, strategies aimed at managing aging frailty are of paramount importance. The guidelines strongly recommend multi-component physical activity as the first-line therapy for the treatment of frailty. For individuals with malnutrition, protein/caloric supplementation is conditionally suggested. However, no specific recommendations were provided for either vitamin D supplementation or hormone-based treatment. Moreover, pharmacological treatment is not recommended [8]. Currently, there are no approved medical therapies available for the management of frailty.

Individuals with aging frailty exhibit phenotypes of weakness, including unintentional weight loss, self-reported exhaustion, slow walking speed, low grip strength and decreased physical performance [3]. It has been hypothesized that the regenerative and differentiating capacity of endogenous stem cells may decline with advancing age, leading to diminished homeostasis and reduced organ functions due to the exhaustion and depletion of endogenous stem cells [6]. Consequently, stem cell therapy emerges as a promising avenue in the treatment of aging frailty [6, 9, 10]. Mesenchymal stem cells (MSCs) have been demonstrated to possess the capability to home to injury sites and exert positive functions in limiting inflammation, stimulating endogenous stem cells, and promoting tissue regeneration [11]. According to multiple sources, MSCs can be categorized into bone marrow-derived MSCs (BM-MSCs), adipose-derived MSCs (ASCs), human umbilical cord-derived MSCs (HUC-MSCs), and so forth [12]. Golpanian et al. [13], and Tompkins et al. [14] have conducted a phase I and a phase II clinical study, respectively, using human allogeneic BM-MSCs intravenously infused for aging frailty. The safety and efficacy endpoints of both trials supported the evidence that intravenous BM-MSCs were safely administered and produced remarkable benefits, improving functional capacity and reducing systematic inflammation among aging frailty patients.

HUC-MSCs are groups of stromal cells presenting in umbilical cord tissues. HUC-MSCs exhibit biological properties of stem cells with self-renewal and multipotency. Compared to BM-MSCs, ASCs and other types, HUC-MSCs have multiple advantages in the field of regeneration medicine. Notably, it is easy to obtain a substantial quantify of HUC-MSCs through successive passages and extensive expansion [15]. Remarkably, HUC-MSCs display immunomodulatory functions and possess anti-inflammatory properties [16, 17]. Furthermore, in vivo, HUC-MSCs demonstrate high proliferation potential, broad differentiation capabilities, low immunogenicity, and a lack of ethical concerns [18–20]. For these reasons, HUC-MSCs has shown therapeutic potential in the treatment of severe or challenging conditions such as graft-versus-host disease [21], multiple sclerosis [22], heart failure [23], type 2 diabetes [24], rheumatoid arthritis [25]. However, up till now there hasn’t been clinical research utilizing HUC-MSCs for the treatment of aging frailty. Based on the current evidence, we conducted a randomized, double-blinded, and placebo-controlled study on aging frailty patients, with the aim of evaluating the safety and efficacy of HUC-MSC transplantation in aging frailty.

## Methods

### Study design

**Fig. 1:**
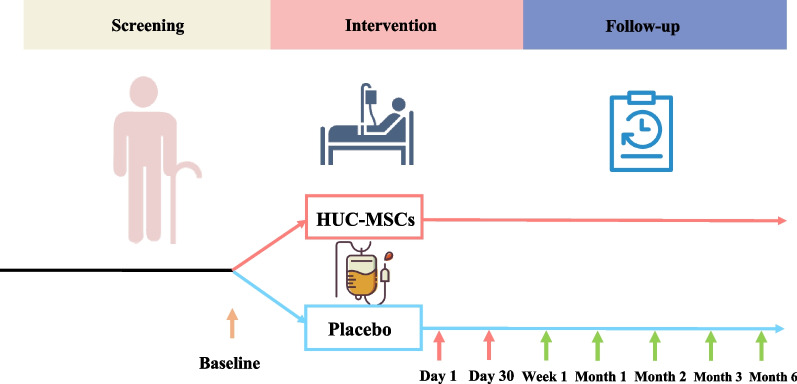
The schedule of study. The intravenous infusions of HUC-MSCs at a dose of 10^6^ cells/kg or placebo were intravenously infused twice at day 1 and day 30. The quality of life, physical function, clinical laboratory parameters and inflammatory cytokines were assessed at baseline and follow-up visits. SAEs and AEs were collected within 6 months following the first treatment

The study was a phase 1/2 randomized, double-blind, placebo-controlled clinical trial. This trial was conducted at the clinical research center of Shanghai East hospital, China between July 3, 2020 (data that first participant enrolled) and January 6, 2022 (date that last participant completed follow-up visit). The study was designed to enroll a total of 30 participants, who will be randomly assigned into the HUC-MSCs treatment group or placebo group. The interventions include intravenous infusions of HUC-MSCs at a dose of 10^6^ cells/kg or placebo once a month for twice. The safety and efficacy assessment will be performed at 1 week, 1 month, 2 months, 3 months, and 6 months after the first treatment (Fig. 1). This trial protocol has been approved by the Human Cell Clinical Research Ethics Committee of Shanghai East Hospital and was supervised by an independent data and safety monitoring board. All participants had provided written informed consent prior to enrollment, as mandated by the Declaration of Helsinki. The study has been registered in ClinicalTrials.gov (NCT04314011).

### Study population

Participants between ages of 60 and 80 years were screened in the communities across Mainland of China. All the participants have provided written informed consent prior to any study procedures. Inclusion criteria for this study were as follows, (1) aged from 60 and 80 years old; (2) meeting the diagnostic criteria of frailty evaluated via the Fried frailty phenotype scale and scored 1–4 [3]; (3) expected to live more than 12 months. Participants were excluded if they had an allergic constitution or positive history of drug allergy, advanced liver disease or renal failure, class III/IV congestive heart failure, myocardial infarction, unstable angina, stroke, uncontrolled hypertension or hyperglycemia, drug or alcohol abuse, had history or presence of malignant tumors, had active autoimmune diseases, had any active infection (including positivity for hepatitis BsAg, hepatitis C antibody, or HIV antibody, or positive PPD test), poor compliance, or planned organ transplantation; a history of participating in another clinical trials within the previous 3 months or surgeries within 6 months, receipt of MSC-based therapy within the previous 4 weeks.

### Screening

In the study, a screening visit was performed after participants gave written informed where a focused medical examination including the assessment of inclusion and exclusion criteria was conducted. After screening, the eligible participants attended a baseline visit and five subsequent 6-month follow-up visits (scheduled at 1-week, 1-month, 2-month, 3-month and 6-month after the first intravenous transplantation of HUC-MSCs). The baseline assessment took place within one month from the screening visit and were completed prior to random assignment and intervention, then the baseline data on demographic, clinical characteristics of participants were collected.

### Randomization

The randomization sequence was obtained using a random number generator by a statistician external to study. A block randomization method with a block size of six was applied to ensure a balanced intergroup assignment. The allocation was sequentially numbered, and the sequence was concealed by sealed opaque envelopes. All the participants were assigned the unique randomization number, and were randomly allocated to the HUC-MSC treatment group or placebo group at a ratio of 1:1 in accordance with randomization sequence. Both clinicians and research assistants were blinded to allocation status.

### Intervention

Eligible participants were randomly assigned in a 1:1 ratio to receive either HUC-MSCs or placebo. In the HUC-MSCs treated group, HUC-MSCs were intravenously infused at a dose of 1 × 10^6^/kg at the fifth passage, and subsequently administrated at 1-month interval. The placebo group participants received the same volume of 0.9% normal saline twice with same intervention procedure. Both products were matched in size, packaging, appearance, and texture.

### Preparation of HUC-MSCs

Clinical-grade HUC-MSCs were obtained from umbilical cord of healthy donors after full-term delivery with the written informed consent. The procedure of processing the samples and culturing HUC-MSCs were conducted fully compliant with current good manufacture practice (GMP) guidelines in GMP laboratory. The graphic workflow illustrating the manufacturing and quality control process for HUC-MSCs is depicted in Additional file 1: Fig. S1. The HUC-MSCs were manufactured as previously described [23, 26, 27]. Briefly, the HUC-MSCs were isolated from umbilical cord tissues which were diced into cubes of approximately 0.5–1.0 cm^3^ following removal of blood and vessels. Subsequently, these tissue cubes were arranged in a tiled fashion and cultured in Alpha-Minimum Essential Medium (α-MEM, Corning) supplemented with 5% UltraGRo-Advanced (Helios). The culture was maintained at 37 °C in a humidified atmosphere of 95% air and 5% CO2 until HUC-MSCs gradually migrated out of the tissue cubes. Following this, the culture medium was refreshed every 2–3 days, and passages of HUC-MSCs were conducted using Trypin-Express (Gibco) solution. The MSCs were cultured and collected up to the fifth passages for intravenous infusion. HUC-MSCs were characterized based on surface markers detected with flow cytometry according to standardized operating procedures [28]. The positive cell surface markers of CD73, CD90, CD105, and negative surface markers of CD11b, CD19, CD31, CD34, CD45, and HLA-DR were confirmed (Additional file 2: Fig. S2A). Moreover, the differentiation capacity of cells was assessed by staining for the detection of chondrogenesis (Safranin O), osteogenesis (Alizarin Red), and adipogenesis (Oil Red O) under specific culture conditions (Additional file 2: Fig. S2B–D). The release criteria of HUC-MSCs also included absence of all tested contaminants (bacteria, mycoplasma, hepatitis B virus, hepatitis C virus, HIV, syphilis, and fungi), endotoxin ≤ 0.5 EU/mL, and a viability ˃90%.

### Study outcomes

The primary efficacy outcome for this study was the general quality of life measured by physical component scores (PCS) of Short Form 36 Health Survey (SF-36) at 6 months after the first intravenous infusion. The secondary efficacy outcomes assessed the overall physical performance which encompassed grip strength, 4-m walking test (4MWT), timed up and go (TUG) test, five times sit to stand test (FTSST), which examined the ability to stand or movement. The serum levels of inflammatory cytokines, including tumor necrosis factor-α (TNF-α), interferon-γ (INF-γ), interleukin-8 (IL-8) and interleukin-17 (IL-17) were also measured and analyzed between the two groups. Sleep quality was assessed via Pittsburgh sleep quality index (PSQI) questionnaire. The mental composite score (MCS) of SF-36 was used to measure the mental health status. The EuroQol visual analogue scale (EQ-VAS) of 0 to 100 were also utilized to assess quality of life in this study, by which the higher scores indicate better health status. The evaluations were conducted at baseline, 1-week, 1-month, 2-month, 3-month, and 6-month follow-up. The safety end point was the difference in the incidence of reported serious adverse events (SAEs) or adverse events (AEs) following first intravenous infusion in the MSCs and placebo arms, including rates of death, thromboembolic events, hospitalization, and significant abnormal laboratory test results.

### Anthropometry

Height in stocking feet and weight in light clothing were measured by the trained staff using a digital tester (Tsinghua Tongfang, China). The grip strength of dominant hand was measured using a hand dynamometry (Tsinghua Tongfang, China). 4MWTs were measured by timing participants walking over 4 m at usual pace. TUG test required participants to stand up from a seated position in a chair (seat height 46 cm), walk 3 m in a straight line, turn around, walk back, and sit down. The time needed to complete TUG test was recorded in seconds (s). FTSST measured the time needed to rise from a seated position and sit down for five repetitions as quickly as possible without using arms.

### Blood samples

At each follow-up visit, about 20 ml peripheral blood samples of every participant were drawn into ethylenediaminetetraacetic acid (EDTA) coated vacuum tubes between 7:00 and 9:00 am. The fresh blood samples were processed within three hours. The samples were centrifuged at 1000 × g for 10 min to separate plasma at 4 °C and then stored at − 80 °C for further analysis using the immunofluorescence assay (Cellgene Biotech, China). The plasma cytokines were detected according to the manufacturer’s instruction. Briefly, 25 μL of capture microsphere antibodies, 25 μL the plasma sample from each patient, and 25 μL of fluorescence detection antibodies were sequentially added to tubes. After fully mixed, the tubes were incubated in the dark at room temperature for 3 h. Subsequently, 1 mL of phosphate buffer solution (PBS) was introduced, followed by centrifugation at 200 g for 5 min. Then, the supernatant from each tube was discarded, and 100 μL of PBS was introduced into the light-shielded tube for subsequent analysis. Afterward, the samples were assessed using a LongCyte™ flow cytometer (Challenbio, China), and the data were analyzed with FCAP Array™ v3.01 (BD Biosciences, USA) software. The fluorescence intensities were converted into corresponding concentrations based on the standard curves generated from serially diluted calibrators for each cytokine.

### Questionnaires

For each participant, participants filled in validated translations of questionnaires that included SF-36, composed of PCS and MCS; the EQ-VAS as well as PSQI regarding quality of life, health and well-being, and sleep quality. Participants were required to fill in the questionnaires at baseline and at each scheduled follow-up visit, and they were required not to change lifestyles during the intervention period.

### Statistical methods

#### Sample size

To determine the required sample size, it was estimated based on a well-established MSCs anti-frailty study conducted by the University of Miami [14]. The sample size for difference between two independent samples of quantitative data was calculated using a two-sided t test and Mann–Whitney test, with a significance level (α) of 0.05 and a power of 80%. A sample size of 15 patients in each group would achieve 80% power to detect a difference in term of effect size (PASS 15.0). We chose to include a total of 30 participants from the community hospital and the outpatient geriatric center to increase the precision of the estimate.

### Statistical analysis

All statistical analyses were based on the intention-to-treat (ITT) patient population and were performed using SPSS (26.0), Prism (9.2.0) and R software (4.1.2). Comparison between two groups at baseline was analyzed using the independent-sample t test or Mann–Whitney U test according to data distribution. Intraindividual comparison of continuous variables at baseline with those at follow-up was performed with paired t test or Wilcoxon rank sum test according to data distribution. For comparisons of effects on various post-treatment evaluations of MSCs treatment, Bonferroni alpha correction was applied, and statistical significance was set as a value of *p* < 0.01. Categorical data were presented as n/N (%) and tested by Chi-square test. The differences between two groups over different time points were analyzed through a mixed effect maximum likelihood regression. Statistical significance was assumed at a value of *p* < 0.05. The safety analysis was performed on ITT population, including all the participants received the treatment in this study.

## Results

### Study population

**Fig. 2:**
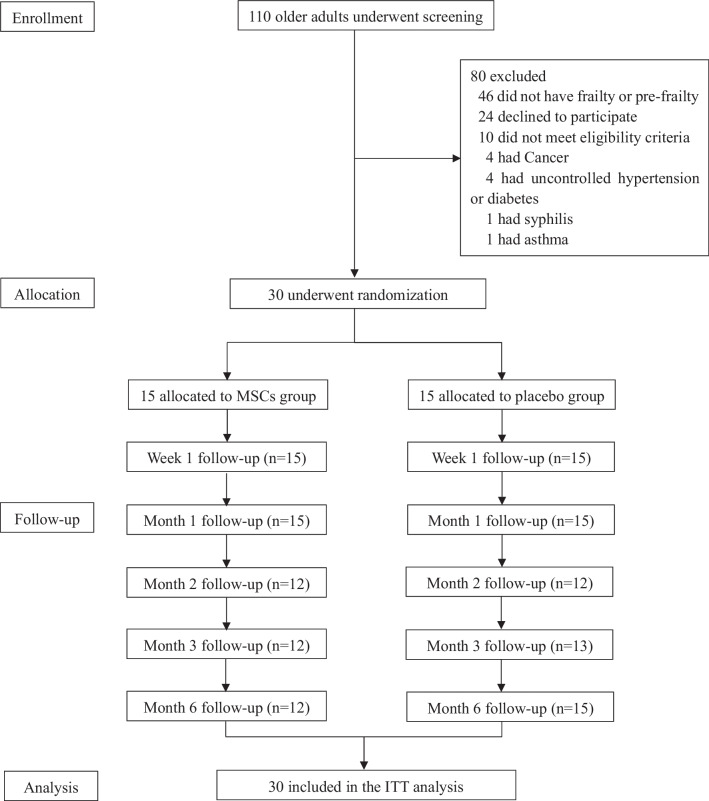
The flow chart of trial. Patient enrollment, allocation, follow-up and analysis

From June 2020 through January 2022, a total of 110 participants were consecutively screened for eligibility in Shanghai East Hospital, and 80 patients were excluded according to the inclusion and exclusion criteria. Finally, 30 patients were randomly assigned in a 1:1 ratio to receive either HUC-MCSs or matching placebo (Fig. 2). Of the thirty participants enrolled in the study, there were 24 participants (12/15 in MSCs group, and 12/15 in placebo group) assessed at month 2, 25 participants (12/15 in MSCs group, and 13/15 in placebo group) at month 3, 27 participants (12/15 in MSCs group, and 15/15 in placebo group) at month 6. One patient in MSCs group was lost to follow-up, who withdrew consent 2 months after the first treatment. Compliance of this trial was excellent with only 14 of scheduled 180 visits (7.77%) missed. The mean age of participants in the MSCs group and placebo group was 67.27 ± 5.23 and 69.27 ± 5.02 years, respectively. At baseline, the two groups showed no statistically significant differences in the demographic and clinical characteristics, including chronic diseases, medications, laboratory tests, physical performance. The baseline demographic characteristics of participants were presented in Table 1.

**Table 1:** Baseline characteristics of participants

Characteristics	MSCs group (N = 15)	Placebo group (N = 15)
Age (years)	67.27 ± 5.23	69.27 ± 5.02
Sex (n, %)		
Male	5 (33.33)	7 (46.67)
Female	10 (66.67)	8 (53.33)
BMI (kg/m^2^)	24.03 ± 2.89	24.95 ± 3.67
Fried frailty phenotype scale	2 (2, 2)	2 (2, 3)
Chronic conditions		
Hypertension (%)	4 (26.67)	7 (46.67)
Dyslipidemia (%)	4 (46.67)	1 (6.67)
Type 2 diabetes mellitus (%)	3 (20)	5 (33.33)
Ischemic cardiomyopathy (%)	2 (13.33)	2 (13.33)
Medication		
Aspirin (%)	2 (13.33)	1 (6.67)
ACEI/ARB (%)	2 (13.33)	4 (26.67)
Calcium antagonists (%)	2 (13.33)	1 (6.67)
Metformin (%)	1 (6.67)	2 (13.33)
Other oral antidiabetics (%)	2 (13.33)	3 (20)
Insulin (%)	2 (13.33)	2 (13.33)
Stain (%)	5 (33.33)	1 (6.67)
Laboratory		
Hemoglobin (g/L)	135.80 ± 7.72	132.47 ± 13.71
White blood cell count (10^9^/L)	5.89 ± 1.37	5.69 ± 1.45
Platelet count (10^9^/L)	203.2 ± 42.75	206.4 ± 73.50
AST (U/L)	23.07 ± 14.26	23.73 ± 8.68
ALT (U/L)	22.73 ± 18.18	21.00 ± 12.62
GFR (mL/min per 1.73 m^2^)	70.73 ± 13.98	75.93 ± 16.35
FBG (mmol/L)	5.94 ± 1.41	5.89 ± 1.26

Data are median (interquartile range, IQR), mean (standard deviation, SD), or n (%)

### Safety

No serious adverse events were observed during the 6 months of the follow-up in this study. The incidenc of adverse events did not differ between the two groups, occurring in 2 (13.33%) participants in the placebo group (one with black pain and one with lower extremity edema) and 1 (6.77%) participant in the MSCs group (experiencing dizziness). All adverse events were transient and considered unrelated to treatment. No patients were withdrawn from this study due to adverse events. There were no clinically important differences between the two groups in the outcomes of laboratory tests and vital signs at all the time points. The summary of adverse events is presented in Table 2.

**Table 2:** Summary of adverse events

Adverse events (n, %)	MSCs (n = 15)	Placebo (n = 15)	P	Related to treatment
Back pain	0 (0)	1 (6.67)	NS	No
Dizziness	1 (6.77)	0 (0)	NS	No
Lower extremity edema	0 (0)	1 (6.67)	NS	No

Data are shown as n (%)

### Clinical outcomes

**Fig. 3:**
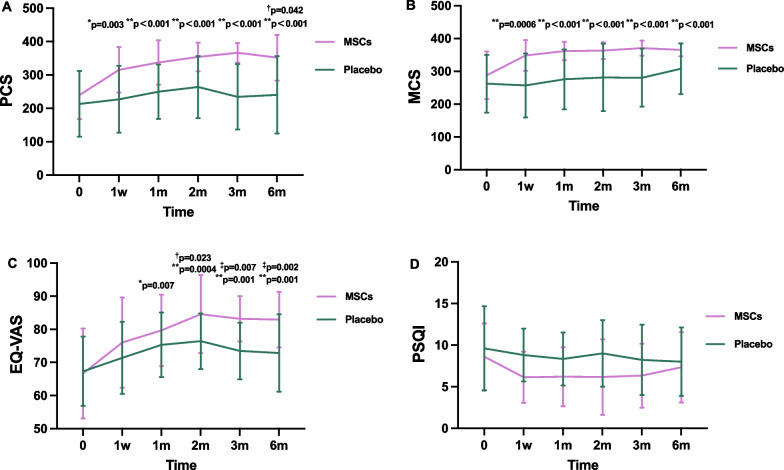
Changes in the scores of qualities of life from baseline to 6-month after intravenous infusion in the MSCs group and placebo group. **A** Physical component scores (PCS); **B** Mental composite score (MCS); **C** EuroQol visual analogue scale (EQ-VAS); **D** Pittsburgh sleep quality index (PSQI). *p < 0.01 vs baseline, **p < 0.002 vs baseline; ^†^*p* < 0.05 vs placebo, ^‡^ *p* < 0.01 vs placebo

Quality of life was evaluated throughout the study and is depicted in Table 3. The primary endpoint was the changes in PCS of SF-36. In comparison with the baseline, there were improvements in the changes of PCS in the MSCs group that began at 1 week of follow-up (+ 75.53 ± 23.02; *p* = 0.003) and continued at 1 month (+ 97.27 ± 23.02; *p* < 0.001) until 6 months (+ 96.41 ± 24.70, *p* < 0.001). The changes in PCS from baseline to month 6 were significantly greater in the MSCs group than in the placebo group (*p* = 0.042) (Fig. 3A). The enhancements were observed in the MCS of SF-36 within the MSCs group, indicating a significant increase from 287.81 ± 72.44 at baseline to 365.29 ± 19.81 at 6 months (*p* = 0.0001), however, there was no statistically significant difference between the MSCs group and the placebo group (Fig. 3B). The EQ-VAS serves as an indicator of perceived quality of life of individuals. In this study, the MSCs group exhibited the enhancement in EQ-VAS scores at the 2-month follow-up (*p* = 0.023) compared to the placebo group, and this improvement persisted until the end of study visit. The most substantial improvements in EQ-VAS were noted at the 6-month follow-up (*p* = 0.002). In the MSCs group, EQ-VAS scores significantly improved compared with baseline, with a group average of 79.67 ± 10.77 at 1 month (*p* = 0.007) and 82.92 ± 8.38 at the end of 6 months (*p* = 0.001). However, the placebo group showed no significant differences in these variables over the 6-month period (Fig. 3C). Additionally, there was no notable difference in the changes of PSQI between the two groups (Fig. 3D).

**Table 3:** Efficacy of endpoints at baseline and follow-up points

Variables	Group	Baseline	1 week	1-month	2-month	3-month	6-month
PCS	MSCs	239.87 ± 72.15*	315.40 ± 68.25**	337.13 ± 66.44**	353.75 ± 42.81**	293.00 ± 153.86**	351.83 ± 68.35**^†^
	Placebo	213.27 ± 98.27	227.07 ± 100.10	249.87 ± 81.22	263.92 ± 92.71	234.46 ± 98.05	240.27 ± 116.08
MCS	MSCs	287.81 ± 72.44**	348.27 ± 47.09**	361.65 ± 27.76**	363.11 ± 25.62**	296.33 ± 154.75**	365.29 ± 19.81**
	Placebo	262.18 ± 88.17	256.76 ± 97.73	275.46 ± 91.12	281.18 ± 102.73	280.32 ± 88.34	307.53 ± 77.12
EQ-VAS	MSCs	66.67 ± 13.58	76.0 ± 13.65	79.67 ± 10.77*	84.58 ± 11.76**^†^	83.17 ± 6.83**^‡^	82.92 ± 8.38**^‡^
	Placebo	67.33 ± 10.50	71.4 ± 10.86	75.33 ± 9.72	76.42 ± 8.39	73.46 ± 8.51	72.87 ± 11.70
PSQI	MSCs	8.60 ± 3.99	6.13 ± 3.07	6.20 ± 3.55	6.17 ± 4.55	6.33 ± 3.85	7.33 ± 4.23
	Placebo	9.60 ± 5.05	8.80 ± 3.17	8.33 ± 3.20	9.0 ± 4.0	8.23 ± 4.23	8.0 ± 4.11
TUG(s)	MSCs	10.20 ± 2.75	8.36 ± 1.70^**†**^	8.25 ± 1.34^†^	7.66 ± 1.46**^†^	7.92 ± 1.35*^†^	7.78 ± 1.63*^†^
	Placebo	10.98 ± 2.46	11.17 ± 3.75	11.05 ± 4.36	11.08 ± 4.66	10.77 ± 3.46	10.97 ± 5.27
4MWT(s)	MSCs	5.04 ± 0.96	4.15 ± 0.69	4.18 ± 0.52*	3.89 ± 0.53**	3.94 ± 0.67**	4.04 ± 0.45**^†^
	Placebo	5.90 ± 1.49	5.82 ± 2.37	5.68 ± 2.46	5.47 ± 2.18	5.68 ± 1.87	6.09 ± 3.39
Grip strength (Kg)	MSCs	17.99 ± 7.31	21.92 ± 7.54	22.27 ± 7.29	23.63 ± 7.13^**†**^	24.96 ± 6.56^†^	25.44 ± 5.44^‡^
	Placebo	17.04 ± 9.65	18.89 ± 9.06	18.76 ± 8.41	17.33 ± 9.28	19.08 ± 9.38	18.33 ± 10.11
FTTST(s)	MSCs	15.10 ± 8.13	11.89 ± 3.41	11.37 ± 3.28	10.33 ± 3.45	10.69 ± 3.47	11.27 ± 3.07
	Placebo	17.37 ± 10.71	17.25 ± 13.67	16.81 ± 13.45	15.68 ± 12.22	16.87 ± 12.26	17.50 ± 9.44
IL-8 (pg/mL)	MSCs	14.30 ± 4.52	12.72 ± 3.14	11.40 ± 4.57	14.27 ± 7.51	9.59 ± 6.06	10.21 ± 3.32
	Placebo	21.29 ± 14.62	15.70 ± 6.28	15.29 ± 5.38	20.28 ± 20.63	13.43 ± 4.72	13.14 ± 6.33
IL-17 (pg/mL)	MSCs	19.25 ± 27.76	12.70 ± 2.63	14.69 ± 6.11	21.82 ± 14.60	17.22 ± 10.06	18.50 ± 22.70^**†**^
	Placebo	11.78 ± 3.36	13.79 ± 3.86	17.66 ± 19.41	19.05 ± 6.70	14.50 ± 4.35	32.76 ± 42.96
IFN-γ(pg/mL)	MSCs	2.70 ± 0.40	3.00 ± 0.34	2.63 ± 0.22	3.10 ± 0.54	3.17 ± 0.73	2.79 ± 0.50
	Placebo	2.60 ± 0.22	3.00 ± 0.38	2.64 ± 0.27	3.19 ± 0.46	3.04 ± 0.64	3.12 ± 0.58
TNFα(pg/m)	MSCs	2.53 ± 0.10	2.59 ± 0.14	2.51 ± 0.04	2.68 ± 0.22	2.76 ± 0.39	2.61 ± 0.25^**†**^
	Placebo	2.53 ± 0.08	2.68 ± 0.20	2.51 ± 0.03	2.73 ± 0.42	2.60 ± 0.25	3.13 ± 1.22

*PCS* Physical component scores; *MCS* Mental composite score; *EQ-VAS* EuroQol visual analogue scale; *PSQI* Pittsburgh sleep quality index; *TUG* Timed Up and Go test; *4MWT* 4-m walking test; *FTTST* Five times sit to stand test; *IL-8* Interleukin-8; *IL-17* Interleukin-17; *INF-γ* Interferon-γ; *TNF-α* Tumor necrosis factor-α

*p < 0.01 vs baseline; **p < 0.002 vs baseline; ^†^*p* < 0.05 vs placebo; ^‡^*p* < 0.01 vs placebo

**Fig. 4:**
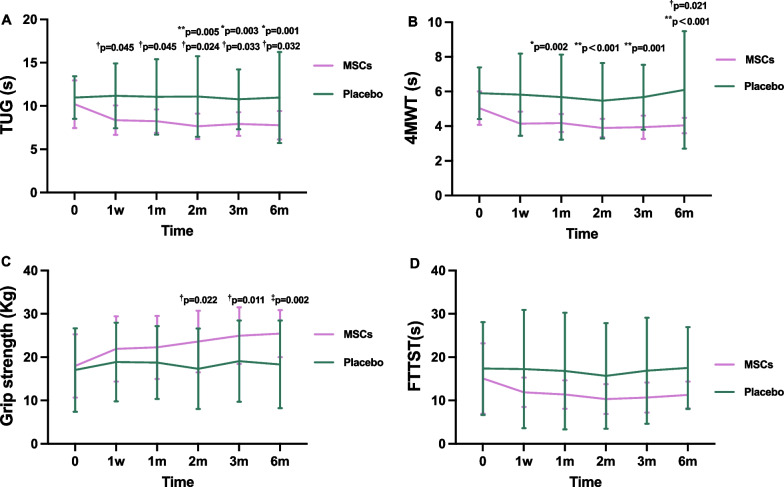
Changes in the levels of physical performance from baseline to 6-month after intravenous infusion in the MSCs group and placebo group. **A** Timed Up and Go (TUG); **B** 4-m walking test (4MWT); **C** Grip strength; **D** Five times sit to stand test (FTTST). *p < 0.01 vs baseline, **p < 0.002 vs baseline; ^†^*p* < 0.05 vs placebo, ^‡^ *p* < 0.01 vs placebo

To compare the functional status between the two groups of patients, we continued to conduct the physical performance tests, including TUG, 4MWT, grip strength, and FTSST at week 1, months 1, 2, 3 and 6 of follow-ups. Notably, compared with the placebo group, greater TUG improvement was observed in the patients injected with HUC-MSCs from the initial visit to all follow-up points (*p* < 0.05)(Fig. 4A). There were substantial improvements in 4MWTs performance in the MSCs group at 6 months (*p* = 0.021) (Fig. 4B). Patients treated with HUC-MSCs experienced enhanced physical performance, as measured by grip strength at several follow-up visits. The most significant increase in grip strength was observed at 6 months of follow-up (*p* = 0.002) (Fig. 4C). However, no statistically significant differences in the FTSST were found for participants tested before, during and at the end of any follow-up points. The physical performance between the two groups of patients is summarized in Table 3.

**Fig. 5:**
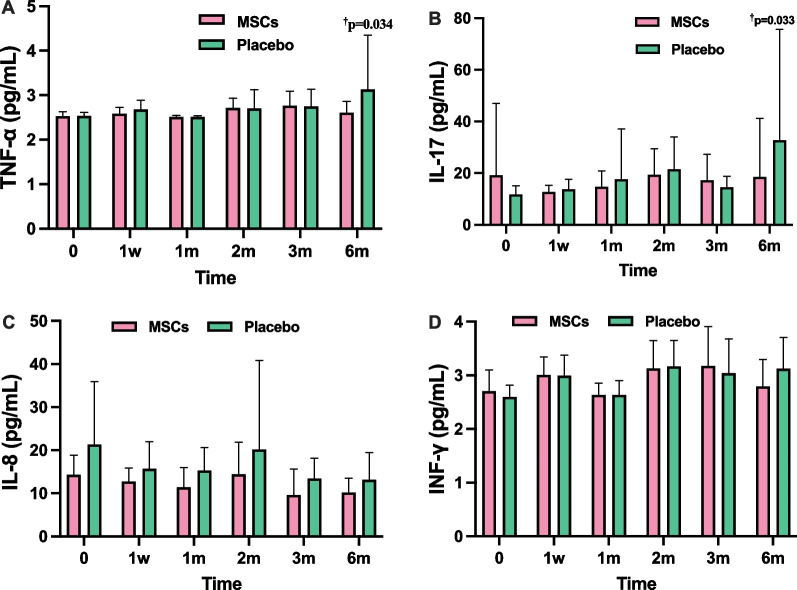
Changes in the levels of inflammatory cytokines from baseline to 6-month after intravenous infusion in the MSCs group and placebo group. **A** Tumor necrosis factor-α (TNF-α); **B** Interleukin-17 (IL-17). **C** Interleukin-8 (IL-8). **D** Interferon-γ (INF-γ). ^†^ *p* < 0.05 vs placebo

It has been reported that the expression of inflammatory cytokines is associated with frailty in the elderly [29]. In this study, the serum levels of cytokines (TNF-α, IL-8, IL-17, INF-γ) were measured six times consecutively in both groups, and the results are presented in Table 3. Of note, in comparison to controls, a decline in the concentrations of TNF-α was observed in patients receiving HUC-MSCs infusion at the month 6 (*p* = 0.034). Consistent with the reduced changes of TNF-α, the levels of IL-17 in the MSC-treated group exhibited a significant decrease compared with the placebo group at month 6 (*p* = 0.033). However, HUC-MSCs did not significantly decrease the levels of IL-8 and IFN-γ at any of the time point during the study (Fig. 5).

## Discussion

This study is the first randomized, double-blind, placebo control clinical trial with intravenous delivery of HUC-MSCs in the elderly individuals with frailty. With an aim to investigate the safety and efficacy of HUC-MSC transplantation in aging frailty, our study has revealed results related to predetermined primary endpoints. HUC-MSCs have been demonstrated to be safe and feasible in the realm of aging-related chronic diseases, as evidenced by the data from various randomized controlled clinical trials [14, 24, 30, 31]. In line with previous studies, intravenously infused HUC-MSCs did not result in any severe adverse events or complications, indicating the safety profile of this novel therapeutic approach. Furthermore, the HUC-MSC treatment in this study induced no adverse immune responses among aging frail individuals, suggesting the well-tolerance and feasibility of MSC-based therapy.

With respect to the primary endpoint defined as the physical component of SF-36 quality of life, our data revealed significant improvements in the PCS scores of SF-36 among individuals receiving MSC treatment. This improvement was observed starting from one week post MSC transplantation and persisted through the final follow-up assessments. Moreover, the administration of HUC-MSCs have led to a substantial improvement in EQ-VAS exclusively at the 2-, 3-, and 6-month follow-up intervals. Furthermore, the mental component of SF-36 quality of life exhibited significant enhancement within the MSCs group during the 6-month follow-up periods. However, there were no significant differences observed between the two groups in terms of sleep quality, as assessed by the PSQI at any of the follow-up time points.

The results of this study suggested that HUC-MSC therapy led to clinically significant improvements in the quality of life and functional performance outcomes. The findings align with previous clinical trials that explored the therapeutic potential of MSCs administration for aging frailty. A phase I clinical trial conducted by Golpanian et al. [13] investigated the effects of intravenous infusion of allogenic BM-MSCs on frail elderly individuals, reporting significant improvements in quality of life, 6-min walk distance, and reduced TNF-α levels. The subsequent phase II study, a randomized, double-blind, placebo controlled clinical trial conducted by Tompkins et al. [14], further demonstrated the efficacy of allogenic BM-MSCs in improving quality of life in older adults with frailty. Collectively, these studies substantiate the potential of MSC-based therapy as a novel approach for ameliorating and preventing the development of aging frailty. As for the physical component of the SF-36 quality of life, which was set as a primary endpoint, our data revealed a significant improvement in the PCS of SF-36 at month 6 in patients receiving HUC-MSCs compared with the placebo group. For patients subjected to HUC-MSCs treatment, the greater PCS were reported starting from one weeks after the procedure and persisted until the end of follow-up period. Furthermore, the MSC treatment led to a significant amelioration in the self-evaluation assessed via EQ-VAS exclusively at the 3- and 6-month follow-ups. In addition to the physical component, the mental composite quality of life was greatly enhanced in the MSCs-treated group during the 2-, 3-, and 6-month follow-up periods. However, in this study, there was no significant difference in the change of PSQI scores between the two groups, indicating that HUC-MSCs did not exert a beneficial effect on ameliorating sleep quality. This finding is consistent with prior research, where patients undergoing the transplantation of hematopoietic stem cells experienced significant sleep disturbances [32]. It is noteworthy that sleep quality is influenced by various external factors, such as environmental conditions, psychological diseases, and lifestyle choices [33], which may impact the response to HUC-MSC infusion in the context of sleep quality. In addition, an extended follow-up period may provide a more comprehensive understanding of therapeutic effects of MSC therapy on sleep quality.

In this study, intravenous administration of MSCs was found to be beneficial for the elderly individuals with frailty. We observed substantial improvements in physical performance capacity following the administration of HUC-MSCs. Specifically, the MSCs group exhibited greater enhancement in grip strength at the 2-, 3- and 6-month follow-up compared to the control group, indicating improved muscle strength in the upper arms. This finding is consistent with a preclinical study utilizing MSCs infusion [34], as well as two clinical studies reported by Golpanian et al. [13] and Tompkins et al. [14]. In addition, the improved performance in TUG tests, assessing the mobility and balance ability [35], demonstrated continuous improvement in patients treated with HUC-MSCs during each post-treatment visit. This finding suggested an overall enhancement in physical function among the MSCs group. Notably, the results indicated an increase in 4MWT performance at the 6-month follow-up in the MSCs group compared to the placebo group. Aging frailty is an aging related condition accompanied by declines in physical capacity, exerting negative effects on the quality of life [36]. The findings of this trial may highlight the effects of HUC-MSCs in ameliorating physical decline associated with aging frailty. However, it is worth noting that there were no significant differences between the two groups in the performance of the FTSST during each follow-up visit. The negative results of FTSST may be attributed to the relatively small sample size of the study population and short duration of follow-up. Consequently, investigations involving larger sample sizes and longer-term follow-up are warranted to elucidate the therapeutic effect of MSC-based therapy on these outcomes among older adults with frailty.

In the present study, we also observed that MSC treatment resulted in the decrease in the levels of TNF-α as well as IL-17 at the 6-month follow-up. However, there were no significant differences in the levels of IL-8 and IFN-γ between the MSCs group and placebo group. Several explanations for our findings warrant consideration. The reduction in the levels of TNF-α and IL-17 following MSC therapy confirmed the anti-inflammatory and immunomodulatory properties of HUC-MSCs. A pervious study has provided evidence supporting the notion that HUC-MSCs can ameliorate cognitive impairment in a mouse model of Alzheimer's disease by modulating neuroinflammation [37]. In addition, as reported by multiple studies, MSCs have been shown to possess anti-inflammatory effects by inhibiting the production of pro-inflammatory cytokines, thereby further attenuating various degenerative and inflammatory disorders, including aging frailty [9, 10, 38]. The declines in both TNF-α and IL-17 levels in older adults treated with HUC-MSCs aligns with existing evidence suggesting that MSCs can alleviate systemic chronic low-grade inflammation, potentially preventing the progression of aging frailty [10, 13, 14]. However, our study did not reveal a significant difference in the levels of IL-8 and IFN-γ between the MSCs and placebo groups. It is well acknowledged that IL-8 and IFN-γ are pro-inflammatory cytokines associated with inflammation and innate immune responses, playing crucial roles in the recruitment, activation and survival of neutrophils at inflammatory sites [39, 40]. The findings of this study may be partially attributed to the small sample sizes. Besides, it is possible that MSCs may exert context-specific effects on signaling pathways to regulate inflammation [41]. Hence, additional investigations are warranted to elucidate the specific mechanisms through which MSCs regulate the production and secretion of pro-inflammatory cytokines. Our findings suggest that intravenous administration of MSCs may alleviate the chronic inflammatory state by reducing the levels of TNF-α and IL-17. However, the potential mechanisms underlying the anti-inflammatory role of MSCs have not been thoroughly elucidated, and further investigations are still needed.

MSCs have been demonstrated to possess regenerative and differentiation properties that contribute to the tissue repair process [42, 43]. Our data in this study indicate that HUC-MSCs may exert their beneficial effects by enhancing physical performance and suppressing chronic inflammation. Furthermore, our study also show that MSC therapy in aging frailty leads to an increased quality of life. In addition to their anti-inflammatory effects, it is conceivable that MSCs have the capability to promote tissue regeneration, muscle strength, and overall physical function. The improved physical performance and enhanced quality of life observed following the administration of HUC-MSCs may be partially attributed to the regenerative capacity of MSCs. The therapeutic benefits of MSC therapy may be derived from the paracrine action of MSCs, including the secretion of growth factors and cytokines, which are involving in modulating the cellular microenvironment and promoting tissue regeneration [44].

Of note, the present study has certain limitations. The relatively short duration of follow-up and the specific characteristics of the study population may impact the observed inflammatory responses. Moreover, as a phase I/II clinical trial, the sample size in our study is limited. Future investigations with larger sample sizes and extended follow-up periods are warranted to validate and further explore the effects of HUC-MSC therapy in aging frailty.

## Conclusions

In conclusion, this randomized controlled clinical trial provides evidence supporting the safety and feasibility of HUC-MSCs therapy for aging frailty. The significant decrease in TNF-α and IL-17, along with the observed improvements in quality of life and physical performance, including TUG tests, grip strength and 4MWT, highlight the potential of HUC-MSCs as a therapeutic option for intervening and preventing of the physical decline associated with aging frailty. Nevertheless, further research is warranted to elucidate the effects of HUC-MSCs therapy on other functional measures and to unravel the potential underlying mechanisms. These findings contribute to the growing body of literature supporting the use of MSC-based interventions in the treatment of aging-related diseases.

### Supplementary Information

**Additional file 1. Supplemental Figure 1.** The graphic flow chart illustrating the manufacturing and assessing processes for clinical grade human umbilical cord-derived mesenchymal stem cells.

**Additional file 2.** **Supplemental Figure 2.** Identification and detection of HUC-MSCs. A. Analysis of flow cytometry showed that HUC-MSCs were positive for the expression of CD73, CD90, CD105, but negative for the expression of CD11b, CD19, CD31, CD34, CD45 and HLA-DR. B. Chondroblast differentiation of HUC-MSCs; C. Adipogenic differentiation of HUC-MSCs; D. Osteogenic differentiation of HUC-MSCs. HUC-MSCs: Human umbilical cord-derived mesenchymal stem cells.

## Data Availability

The dataset used and analyzed during the current study is available from the corresponding author on reasonable request.

## Abbreviations

HUC-MSCs: Human umbilical cord-derived mesenchymal stem cells
SAEs: Serious adverse events
AEs: Adverse events
PCS: Physical component scores
MCS: Mental component scores
SF-36: Short Form 36 Health Survey
EQ-VAS: EuroQol visual analogue scale
PSQI: Pittsburgh sleep quality index
TUG: Timed up and go
4MWT: Four-meter walking test
FTSST: Five times sit to stand
TNF-α: Tumor necrosis factor-α
INF-γ: Interferon-γ
IL-8: Interleukin-8
IL-17: Interleukin-17
