# Supplementary material for: Safety and efficacy of umbilical cord tissue-derived mesenchymal stem cells in the treatment of patients with aging frailty: a phase I/II randomized, double-blind, placebo-controlled study
Source: Stem Cell Res Ther. 2024 Apr 29;15:122. doi: 10.1186/s13287-024-03707-2 (PMC11057094; doi:10.1186/s13287-024-03707-2)
Supplement: Supplementary file 1 — Additional file 1. Supplemental Figure 1. The graphic flow chart illustrating the manufacturing and assessing processes for clinical grade human umbilical cord-derived mesenchymal stem cells. [file 13287_2024_3707_MOESM1_ESM.pdf]

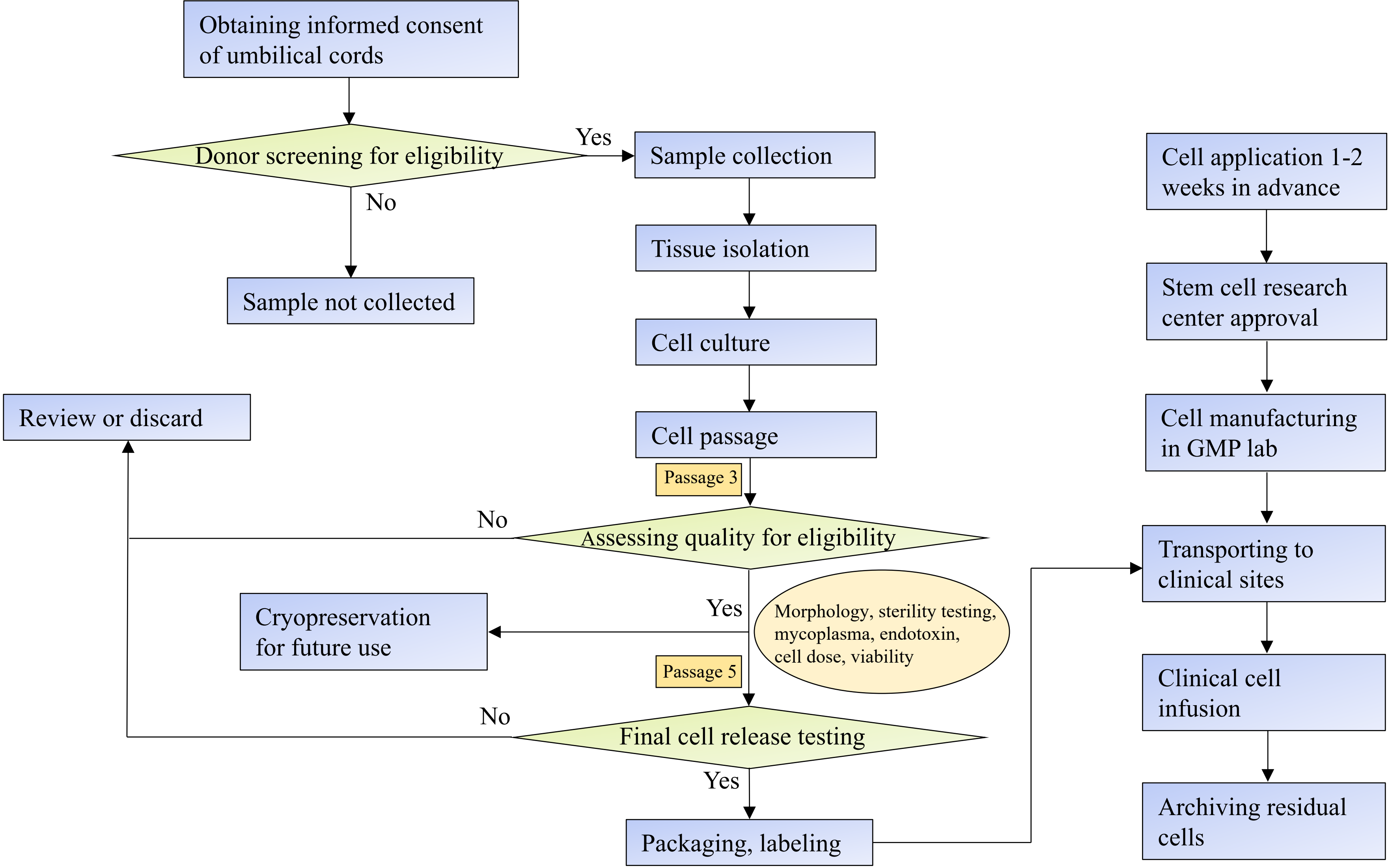

Supplemental Figure 1. The graphic flow chart illustrating the manufacturing and assessing processes for clinical-grade human umbilical cord-derived mesenchymal stem cells.
