# Supplementary material for: Safety and efficacy of umbilical cord tissue-derived mesenchymal stem cells in the treatment of patients with aging frailty: a phase I/II randomized, double-blind, placebo-controlled study
Source: Stem Cell Res Ther. 2024 Apr 29;15:122. doi: 10.1186/s13287-024-03707-2 (PMC11057094; doi:10.1186/s13287-024-03707-2)
Supplement: Supplementary file 2 — Additional file 2. Supplemental Figure 2. Identification and detection of HUC-MSCs. A. Analysis of flow cytometry showed that HUC-MSCs were positive for the expression of CD73, CD90, CD105, but negative for the expression of CD11b, CD19, CD31, CD34, CD45 and HLA-DR. B. Chondroblast differentiation of HUC-MSCs; C. Adipogenic differentiation of HUC-MSCs; D. Osteogenic differentiation of HUC-MSCs. HUC-MSCs: Human umbilical cord-derived mesenchymal stem cells. [file 13287_2024_3707_MOESM2_ESM.pdf]

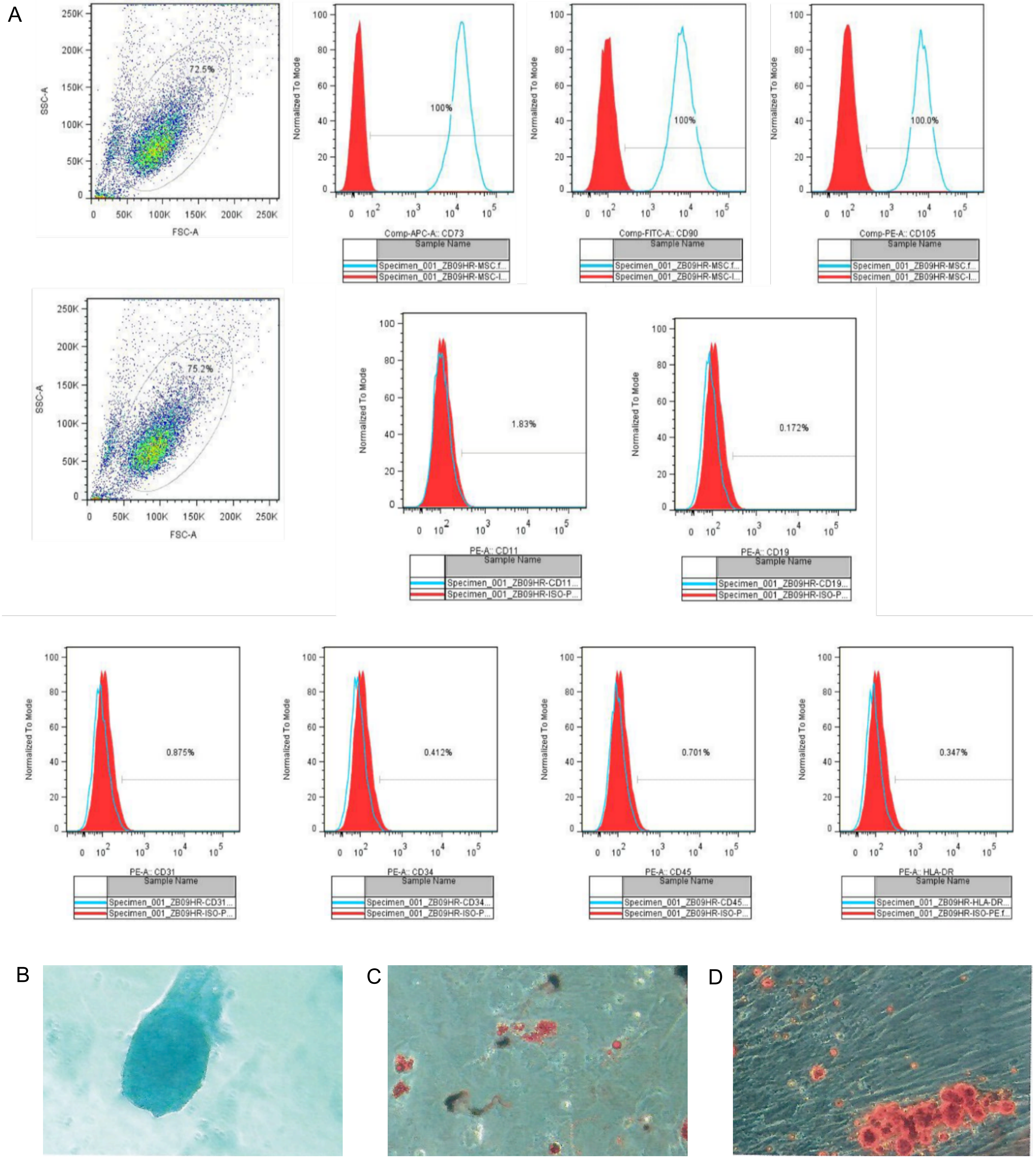

Supplemental Figure 2. Identification and detection of HUC-MSCs. A. Analysis of flow cytometry showed that HUC-MSCs were positive for the expression of CD73, CD90, CD105, but negative for the expression of CD11b, CD19, CD31, CD34, CD45 and HLA-DR. B. Chondroblast differentiation of HUC-MSCs (× 10); C. Adipogenic differentiation of HUC-MSCs (× 20); D. Osteogenic differentiation of HUC-MSCs (× 20). HUC-MSCs: Human umbilical cord-derived mesenchymal stem cells.
